# Similar Population of CD133+ and DDX4+ VSEL-Like Stem Cells Sorted from Human Embryonic Stem Cell, Ovarian, and Ovarian Cancer Ascites Cell Cultures: The Real Embryonic Stem Cells?

**DOI:** 10.3390/cells8070706

**Published:** 2019-07-11

**Authors:** Irma Virant-Klun, Petra Skerl, Srdjan Novakovic, Eda Vrtacnik-Bokal, Spela Smrkolj

**Affiliations:** 1 Reproductive Division, Department of Obstetrics and Gynaecology, University Medical Centre Ljubljana, 1000 Ljubljana, Slovenia; 2 Department of Molecular Diagnostics, Institute of Oncology Ljubljana, 1000 Ljubljana, Slovenia; 3 Gynecological Division, Department of Obstetrics and Gynecology, University Medical Centre Ljubljana, 1000 Ljubljana, Slovenia

**Keywords:** human, very small embryonic-like stem cells (VSELs), embryonic stem cells, ovary, ovarian cancer ascites, CD133, DDX4, magnetic-activated cell sorting, differentiation

## Abstract

A population of small stem cells with diameters of up to 5 μm resembling very small embryonic-like stem cells (VSELs) were sorted from human embryonic stem cell (hESC) cultures using magnetic-activated cell sorting (MACS) based on the expression of a stem-cell-related marker prominin-1 (CD133). These VSEL-like stem cells had nuclei that almost filled the whole cell volume and expressed stem-cell-related markers (CD133, SSEA-4) and markers of germinal lineage (DDX4/VASA, PRDM14). They were comparable to similar populations of small stem cells sorted from cell cultures of normal ovaries and were the predominant cells in ascites of recurrent ovarian cancer. The sorted populations of CD133+ VSEL-like stem cells were quiescent in vitro, except for ascites, and were highly activated after exposure to valproic acid and follicle-stimulating hormone (FSH), indicating a new tool to study these cells in vitro. These VSEL-like stem cells spontaneously formed clusters resembling tumour-like structures or grew into larger, oocyte-like cells and were differentiated in vitro into adipogenic, osteogenic and neural lineages after sorting. We propose the population of VSEL-like stem cells from hESC cultures as potential original embryonic stem cells, which are present in the human embryo, persist in adult human ovaries from the embryonic period of life and are involved in cancer manifestation.

## 1. Introduction

A population of small stem cells showing pluripotency persists from the embryonic period of life in adult human tissues and organs, such as bone marrow, umbilical cord blood and peripheral blood, and are termed very small embryonic-like stem cells (VSELs). These cells were first discovered by the research group of Ratajczak [1,2] and further explored by several other groups in bone marrow [3,4,5,6,7,8], umbilical cord blood [9,10,11,12], peripheral blood [13,14], uterine endometrium [15], testis [16], retina [17] and bone [18] in humans and rodents. VSELs are proposed to originate in the embryonic epiblast during development [19,20], mobilized into the peripheral blood under inappropriate conditions (e.g., stroke, myocardial ischaemia, skin burn injury, septic shock, brain injury, Crohn’s disease) to regenerate the tissues and organs [21,22,23,24,25,26], and proposed to be able to regenerate organs, such as the pancreas, brain, lung, liver or heart, by transplantation [27,28,29,30,31]. Under inappropriate conditions in the body, these cells may form tumours [32] and are involved in the manifestation of ovarian cancer. In general, VSELs are present in a body at a dormant state and are activated when needed [33,34]. They strongly express several markers, including prominin-1 (CD133), a marker of stem cells (e.g., haematopoietic stem cells, endothelial progenitor cells, glioblastoma cells, neuronal and glial stem cells, etc.), which is a useful cell surface marker of VSELs and possibly acts as an organiser of the cell membrane topology. Antibodies against this molecule, conjugated to paramagnetic beads or fluorochromes, are powerful biological tools for the isolation of VSELs from adult human tissues [35,36]. Furthermore, this marker is commonly used to isolate cancer stem cells from different types of tumours, including ovarian tumours [37,38,39,40].

Similar to other tissues and organs, some studies clearly showed that VSELs are also present in adult human ovaries. Our group first found VSEL-like stem cells in the ovarian surface epithelium of women with no naturally present follicles and oocytes, postmenopausal women and patients with premature ovarian failure [41,42,43,44]. These cells developed into oocyte-like cells in vitro [41,42,45,46]. These findings were further confirmed by other research groups in humans [47,48] and other mammalian species, such as mice [47], monkeys [47], sheep [47,49], and pigs [50]. However, these cells were confirmed to be involved in the manifestation of borderline ovarian cancer [51] and were found in situ in ovarian sections of patients with serous ovarian carcinoma [52].

Small VSELs possess an embryonic character because they originate from the embryo [20]. The embryonic character of these cells and their relation to the germinal lineage (i.e., expression of marker DDX4/VASA) is still poorly understood; therefore, the aim of this study was to determine whether VSEL-like stem cells are also present in a culture of human embryonic stem cells and to sort them using CD133-based magnetic-activated cell sorting (MACS) compared to cell cultures of healthy, non-malignant ovaries and ascites from recurrent ovarian cancer to prove their embryonic character.

## 2. Materials and Methods

### 2.1. Materials and Experimental Design

This study was approved by the Slovenian Medical Ethical Committee (Ministry of Health of the Republic of Slovenia, No. 135/09/09 and 154/07/10) and conducted in accordance with the Declaration of Helsinki. The small, VSEL-like stem cells were comparatively analysed in two cell cultures of hESCs, two cell cultures of healthy (non-malignant) ovarian tissue (two women, aged 39 and 42 years; one with primary ovarian insufficiency and with a number of residual follicles in her ovaries, and another with ovarian endometrioma and normal folliculogenesis/oogenesis in her ovaries with a mass of vital follicles at different stages of growth), and five cell cultures from ascites (five women, aged 66, 81, 58, 58 and 69 years) with recurrent ovarian cancer before and after CD133-based MACS selection. The healthy ovarian tissue was retrieved via a biopsy of a small piece (volume of 5 mm^3^) of ovarian cortex, while in patients with recurrent ovarian cancer their ascites was retrieved under sterile conditions, centrifuged, and cells where further processed for this research. The ovarian tissue and ascites samples were retrieved after written informed consent was obtained from the donating women. The frozen culture of hESCs (HS999 p15) for this research was kindly provided by the Karolinska Institute, Stockholm, Sweden (Prof. Outi Hovatta).

Cells were analysed for the expression of stem cell and germinal lineage-related markers using immunocytochemistry and quantitative polymerase chain reaction (qPCR) analysis after sorting, expanded in vitro using valproic acid and follicle-stimulating hormone (FSH) and in vitro differentiated into adipogenic, osteogenic and neural lineages using established protocols.

### 2.2. Cell Cultures

The thawed hESCs were cultured in Dulbecco’s Modified Eagle Medium/Nutrient Mixture F-12 (DMEM/F-12) culture medium, including 20% KnockOut Serum Replacement (Gibco Life Technologies, Carlsbad, CA, USA), 1 mM l-glutamine (PAA, Pasching, Austria), 1% nonessential amino acids (PAA), 0.1 mM 2-mercaptoethanol (Invitrogen Life Technologies, Carlsbad, CA, USA), 13 mM hydroxyethyl piperazineethanesulfonic acid (HEPES), and 4 ng/mL human basic fibroblast growth factor (FGF) (Sigma-Aldrich, St. Louis, Misuri, USA) and without feeder cells. After thawing, the hESC culture was established for 1 week and then used for cell sorting.

For ovarian cell cultures, the ovarian tissue was first enzymatically digested in two enzyme solutions. After mincing the biopsies using a sterile scalpel, they were incubated in 0.7 mg/mL collagenase, type XI (Sigma-Aldrich), for 15 min at 37 °C and centrifuged for 8 min at 1400 rpm (300× *g*). Then, the supernatant was discarded, and the pellet was resuspended in a 1:1 mixture of hyaluronidase (80 I.U./mL, SynVitro Hydase, Origio, Cooper Surgical, Målov, Denmark) and 0.7 mg/mL collagenase, type XI. After 15 min of incubation in this mixture at 37 °C, 10% foetal bovine serum (FBS, Gibco) was added to block the enzymatic activity, and the sample was again centrifuged for 8 min at 1400 rpm. The supernatant was then discarded, and the cells were washed in the next step with a basal medium consisting of DMEM/F-12 culture medium (Sigma-Aldrich) with 3.7 g/L NaHCO_3_ and 1% penicillin/streptomycin (Sigma-Aldrich), with the pH adjusted to 7.4 using 1 M NaOH. After washing, the cells were resuspended in DMEM/F-12 with 20% FBS and passed through a 70-μm cell strainer (BD Falcon, Franklin Lakes, New Jersey, USA) to remove larger particles. The filtered cells were then seeded into culture plates and cultured in DMEM/F-12 culture medium with 3.7 g/L NaHCO_3_, 1% penicillin/streptomycin (Sigma-Aldrich), pH adjusted to 7.4 (with 1 M NaOH), and 20% (*v*/*v*) foetal bovine serum (FBS). Separately, the ovarian follicles/oocytes were isolated from the minced ovarian tissue before setting up the cell culture and staining using Trypan Blue or Neutral Red to estimate their vitality. After staining with Trypan Blue, the live cells did not stain, while the dead cells stained blue. On the other hand, the live cells stained red after Neutral Red staining, while the dead cells did not stain. 

Both hESCs and ovarian cells were cultured in four-well culture plates in a CO_2_ incubator at 37 °C and 6% CO_2_ in air. In each culture, the cells and colonies that they formed were monitored daily under a heat-stage-equipped inverted microscope with Hoffman illumination and at 200× to 1000× magnification (Eclipse T-2000; Nikon, Tokyo, Japan, equipped with a Nikon Digital Sight Camera).

For culture of cells from ovarian cancer ascites, 1000 mL of ascites was used to collect cancer stem cells following the procedures described by Latifi et al. [53]. Briefly, ascites was centrifuged at 600 rpm for 5 min to collect the cells. Then, the cells were washed three times with a PBS buffer. Contaminating red blood cells in the cell pellet were removed by resuspending the cells in a red blood cell lysis buffer (150 mM ammonium chloride, 10 mM potassium bicarbonate and 1 mM ethylenediaminetetraacetic acid (EDTA; ACK), followed by incubation for 3 to 5 min at room temperature. Ascites cells were concentrated by centrifuging at 300 rpm for 5 min. The cells were washed twice with PBS and resuspended in Roswell Park Memorial Institute (RPMI) medium supplemented with 5 μg/mL insulin, 20 ng/mL ethylene glycol (EG), 10 ng/mL basic fibroblast growth factor (bFGF), 1% FBS, and penicillin/streptomycin. In the next step, cells were seeded on ultralow attachment plates (Costar 24 Well Plate, with ultralow attachment surface, Corning, New York, NY, USA), and maintained in a CO_2_ incubator at 37 °C in the presence of 6% CO_2_ in air. Under these conditions, non-adhering (NAD) cells floated as spheroids, which are characteristic formations of (ascites) cancer stem cells, while adhering (AD) non-cancer cells attached to the plate.

In each cell culture, the proportion of VSEL-like stem cells in at least ten random fields under the microscope prior to cell sorting was estimated. The mean proportion of VSEL-like stem cells per field and the proportion of VSEL-like stem cells per all counted cells were evaluated in each cell culture and were compared between different types of cell cultures prior to cell sorting. Statistical analysis was performed using a t-test and a chi-square test with a statistical significance set at *p* < 0.05. 

Human adult dermal fibroblasts (Cascade Biologics, Thermo Fisher Scientific, Waltham, MA, USA, Cat. No. C-013-5C) were cultivated in parallel as a negative control for our experiments. 

### 2.3. Magnetic-Activated Cell Sorting Based on CD133 Expression

*Preparation of cells*: The MACS procedure was performed approximately one week after the establishment of each cell culture using the Miltenyi Biotec (Bergisch Gladbach, North Rhine- Westphalia, Germany) tools. First, the number of cells was evaluated. The suspension of cells without cell clusters, which was previously filtered through the 30-μm Pre-Separation Filters (Cat. No. 130-041-407), was centrifuged for 10 min at 1400 rpm (300× *g*), and the supernatant was completely removed. The cell pellet was resuspended in 60 μL of separation buffer (for ≈10^7^ cells), which was freshly prepared via dilution (1:20) of MACS Bovine Serum Albumin (BSA) Stock Solution (Cat. No. 130-09-376) with autoMACS Rinsing Solution (Cat. No. 130-091-222). Then, the volume of 20 μL of FcR Blocking Reagent (human; Cat. No. 130-059-901) was added. In the next step, 20 μL of CD133 MicroBead Kit-Tumor Tissue (human; Cat. No. 130-100-857) reagent was added. The cell suspension was mixed and incubated for 15 min in a refrigerator (2–8 °C) with permanent mixing. Next, the cells were washed by adding 1–2 mL of separation buffer and centrifuged at 1400 rpm for 10 min. The supernatant was completely removed. This step was followed by staining of the cells with the addition of 10 μL of Labeling Check Reagent-PE (Cat. No. 130-095-228), mixing, and incubation of cells in the refrigerator for 5 min (in the dark). After this, the cells were washed with 1–2 mL of separation buffer and centrifuged at 1400 rpm for 10 min. The supernatant was completely removed, and the cell pellet was resuspended in a volume of 500 μL of separation buffer.

*Magnetic separation*: The column was placed near the magnet and washed with 500 μL of separation buffer. The suspension of cells was placed at the top of the column. The fraction of non-labelled cells that passed the column without binding was stored. Then, the column was washed three times with 500 μL of separation buffer, and non-labelled cells were combined with cells from the previous step. A fresh centrifuge tube was placed below the column, and the column was removed from the magnet. A volume of 1 mL of separation buffer was added to the top of the column, and the labelled cells were gently pushed from the column into a sterile dish. If the population of labelled cells was not pure, the suspension of these cells was gently pushed through the fresh column again. A small portion of the suspension of sorted cells was observed under a fluorescence microscope to assess the labelling (red fluorescence) of cells, while the majority of cell suspension was used for a further culture. After each MACS-sorting, the labeling of CD133+ and CD133− cell populations was tested via the addition of a Labeling Check reagent-PE and observation under a fluorescence microscope. The proportion of labeled cells in each cell population was evaluated and compared to another population. It was found that in the CD133+ cell population, most cells (up to 90%) were labeled with CD133 marker (Supplemental Figure S2), while only rare cells (up to 5%) in the CD133− cell population were labeled with this marker. In addition, the proportion of VSEL-like stem cells per all cells was estimated in each CD133+ cell population (at least ten random fields under the microscope). 

### 2.4. Exposure of Sorted Cells to Valproic Acid and FSH for Activation

The VSEL-like stem cells, which were sorted from cell cultures, were maintained in a CO_2_ incubator (37 °C, 6% CO_2_ in air) in DMEM/F-12 culture medium containing 10% FBS and 1 mM valproic acid (VPA; Sigma-Aldrich, P6273-100 mL; 1 μL VPA per 5 mL of medium) and 10 U/mL of recombinant FSH (Puregon, Merck Sharp & Dohme B.V., Haarlem, The Netherlands) without feeder cells for up to 8 months. The culture medium was refreshed every 7 days.

### 2.5. Immunocytochemistry for CD133, DDX4, SSEA4, PRDM14, and S100 Expression

Immunofluorescence: It was used to detect the expression of the markers CD133 and DDX4 in cells. The analysed cells were fixed in 4% paraformaldehyde, permeabilised with 0.2% Triton (for DDX4) and incubated for 20 min with 10% FBS. Then, the cells were incubated for 1 h at room temperature with polyclonal rabbit anti-DDX4 primary antibodies (diluted 1:100, AB4330, Merck Millipore, Burlington, MA, USA) or mouse anti-CD133/1 (AC133) monoclonal antibodies (diluted 1:100, Cat. No. 13-090-422, Miltenyi Biotec) in the dark and after washing for 30 min with polyclonal goat anti-rabbit immunoglobulin biotinylated secondary antibodies (diluted 1:200; Dako, Glostrup, Denmark) or with polyclonal rabbit anti-mouse immunoglobulin biotinylated secondary antibodies (1:200; Dako). After washing with phosphate buffered saline (PBS), the cells were mounted using Vectashield mounting medium with 4′,6-diamidino-2-phenylindole (DAPI) to stain the genetic material blue and observed under a fluorescence microscope. For a negative control, the primary antibodies were deleted from the procedure and replaced with 1% FBS. Stained cells were monitored under a fluorescence microscope, and the positively stained cells expressed red fluorescence for DDX4 and green fluorescence for CD133.

3,3′-Diaminobenzidine (DAB) procedure: Cells were fixed in 4% paraformaldehyde, permeabilised with 0.2% Triton and incubated with 3% H_2_O_2_ for 10 min to block the endogenous peroxidase activity, and for 20 min with 10% FBS to block the nonspecific binding sites in cells. In the next step, the cells were incubated for 1 h at room temperature with mouse anti-CD133 primary antibody (diluted 1:100, Miltenyi Biotec, Cat. No. 130-090-422), rabbit anti-DDX4 primary antibody (diluted 1:100, Millipore, Burlington, MA, USA, Cat. No. AB4330), rabbit anti-PRDM14 primary antibody (diluted 1:100, Abcam, Ab187881, Cambridge, U.K.), mouse anti-SSEA-4 monoclonal antibody (diluted 1:100, Millipore), or rabbit anti-S100 polyclonal antibodies (1 : 500, DakoCytomation, Glostrup, Denmark). After washing with PBS, the cells were incubated at room temperature with biotinylated secondary antibodies, polyclonal goat anti-rabbit immunoglobulins (diluted 1:600; DakoCytomation) or polyclonal rabbit anti-mouse immunoglobulins (diluted 1:400; DakoCytomation) for 30 min and then with an ABC reagent (Vectastain ABC Kit-Standard; Vector Laboratories, Burlingame, CA, USA) for 30 min. At the end, the cells were incubated in a DAB substrate (Sigma-Aldrich) until brown staining (“positivity”) appeared but no longer than 5 min, washed with PBS and observed under an inverted microscope (Hoffman illumination) to detect positive, brown-stained cells or cell colonies. For a (technical) negative control, the primary antibodies were deleted from the procedure and replaced with 1% FBS. In addition to the studied cell cultures, the DAB method was also performed on human adult dermal fibroblasts for a negative control. 

### 2.6. qPCR Analysis of CD133 and DDX4 Expression

RNA isolation: Total RNA was isolated from samples using a MiRNeasy Mini kit (Qiagen, Hilden, Germany) according to the manufacturer’s instructions.

qPCR: RNA transcription (300 ng) into cDNA was performed using a High-Capacity cDNA Reverse Transcription kit (Applied Biosystems, Thermo Fisher Scientific, Waltham, MA, USA). The expression of target genes was followed using TaqMan chemical methods on an ABI7900 machine (Applied Biosystems) for quantitative PCR in real time (qPCR). For an endogenous control, the housekeeping gene *GAPDH* (*glyceraldehyde-3-phosphate-dehydrogenase*) was used. The assays Hs01009259_m1 for the gene *PROM1/CD133*, Hs00987125_m1 for the gene *DDX4/VASA* and Hs99999905_m1 for the gene *GAPDH* were used.

Cells from the human colon cancer cell line HT-29, established and maintained at the Oncological Institute Ljubljana, were used as a positive control, and human adult dermal fibroblasts provided by Cascade Biologics (Thermo Fisher Scientific, Cat. No. C-013-5C) were used as a negative control. Each sample was analysed in triplicate, and the entire analysis was repeated two times. The data were analysed with Sequence Detection System v2.4 (SDS2.4), an upgrade of PCR system (Applied Biosystems, Thermo Fisher Scientific, Waltham, MA, USA) to obtain the Ct values. The level of expression of each target gene was expressed as a fold change after normalisation to a normal ovary. A more than two-fold change in gene expression compared to the normal ovary sample was considered to be up- or down-regulation of a specific gene.

### 2.7. In Vitro Differentiation of Cells into Adipogenic, Osteogenic and Neural lineages

Adipogenic lineage: For adipogenic differentiation, the sorted cells were cultured in a medium consisting of hESC medium (DMEM/F12, 20% KnockOut Serum Replacement (Gibco, Thermo Fisher Scientific), 1 mM l-glutamine (PAA), 1% non-essential amino acids (PAA), 0.1 mM 2-mercaptoethanol (Invitrogen), 13 mM HEPES, 8 ng/mL human basic fibroblast growth factor (bFGF, Sigma-Aldrich, St. Louis, MO, USA), and 1% penicillin/streptomycin) supplemented with 20% follicular fluid serum from the in vitro fertilisation programme. The differentiation medium was changed every 3–4 days. To visualize the intracytoplasmic lipid droplets, the cell culture was assessed with Oil Red O staining. After 2 weeks of differentiation, the cell culture was fixed in 4% paraformaldehyde (PFA) for 20 min and incubated for 10 min in an Oil Red O work solution. After thorough washing, the cells were observed under an inverted microscope (Hoffman illumination) for the presence of lipid droplets, which were stained red.

Osteogenic lineage: Osteogenic differentiation of cell cultures after sorting was performed in a medium consisting of low glucose DMEM, l-glutamine, FBS, dexamethasone (Sigma-Aldrich), l-ascorbic acid 2-phosphate (Sigma-Aldrich), β-glycerophosphate (Sigma-Aldrich) and penicillin/streptomycin. To evaluate the cell differentiation potential, von Kossa staining was performed to visualise the calcium accumulation after 4 weeks of exposure of cells to osteogenic differentiation medium. The cells were fixed in 4% PFA, incubated in 2% silver nitrate in the dark, washed with double-distilled water and exposed to UV light for 25 min. After washing, cells were monitored under an inverted microscope (Hoffman illumination) to identify black stained calcium deposits.

Neural lineage: Cells from cultures after sorting were cultured in DMEM/F12 culture medium supplemented with 1% human serum albumin (HSA), 80 ng/mL human basic FGF, 30 μM forskolin, 1% nonessential amino acids, 0.1 mM 2-mercaptoethanol, and 1% Insulin-Transferrin-Selenium (ITS). The cells were monitored daily, and after the first morphological changes, they were stained using immunocytochemistry for S100 expression.

The cell culture of human adult dermal fibroblasts was used as a negative control.

## 3. Results

### 3.1. VSEL-Like Stem Cells in Cell Cultures

**Figure 1:**
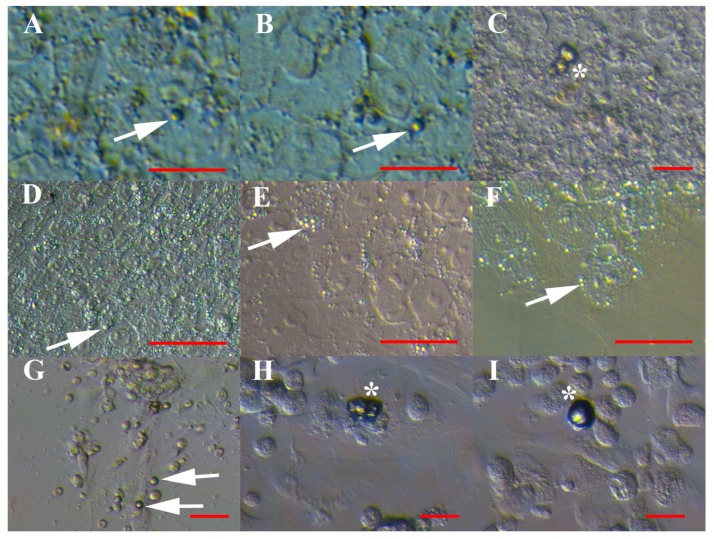
VSEL-like stem cells with diameters of up to 5 μm in cell cultures as individual cells (arrows) or cell clusters (asterisks). (**A**–**F**) Human embryonic stem cells (hESCs), and (**G**–**I**) normal ovary cells. Inverted microscope (Hoffman illumination). Red bar: 10 μm for A–C, G–I; 50 μm for D–F.

**Figure 2:**
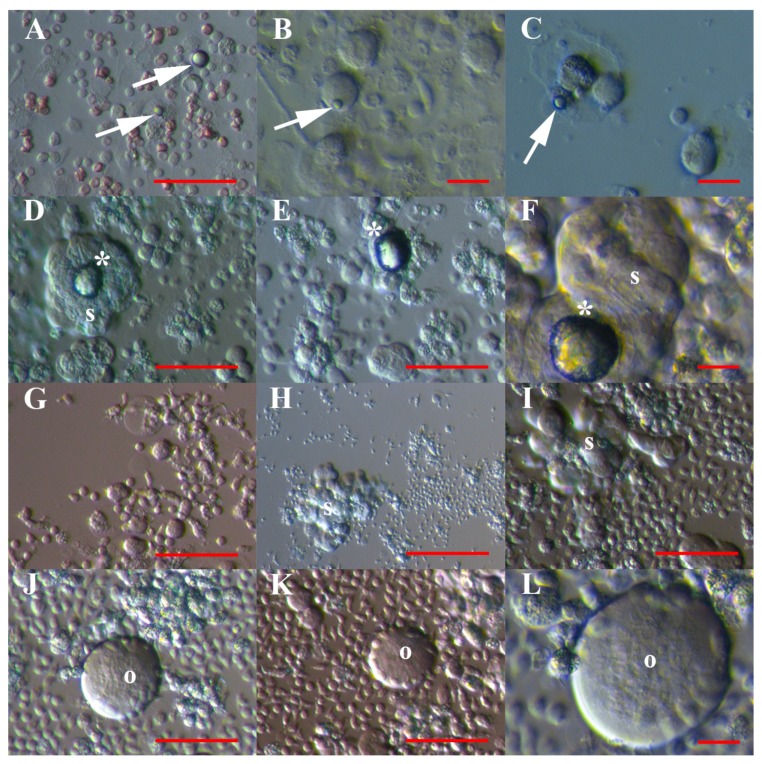
VSEL-like stem cells (arrows) with diameters of up to 5 μm in cell cultures from recurrent ovarian cancer ascites. (**A**–**C**) VSEL-like stem cells among other types of cells in a cell culture; (**D**–**F**) VSEL-like stem cells forming cell clusters (asterisks) attached to spheroids; (**G**–**I**) highly proliferating VSEL-like stem cells growing into larger cells and forming spheroids; and (**J**–**L**) highly proliferating VSEL-like stem cells growing into larger, oocyte-like cells. Inverted microscope (Hoffman illumination). Legend: s—spheroid; o—oocyte-like cell. Red bar: 10 μm for B, C, F, L; 50 μm for A, D, E, G, I–K; 100 μm for H.

In cell cultures of hESCs, normal (non-malignant) ovary cells and recurrent ovarian cancer ascites, we noticed a similar population of typical, small and round cells with diameters of up to 5 μm resembling VSELs (see Figure 1 and Figure 2). They appeared as small “metal beads”, which were slightly yellow, shining and attached to other types of cells at the dish bottom or floating in a culture medium. A proportion of these VSEL-like stem cells grew into larger oogonial-like cells with diameters of approximately 10 μm. As seen in Table 1, the population of VSEL-like stem cells comprised a relatively low proportion of all cells in cultures of hESCs (Figure 1A–F) and normal ovaries (Figure 1G–I) and formed comparable yellow shining cell clusters resembling tumour-like structures in both types of cell cultures (Figure 1C,H,I). In general, these typical VSEL-like stem cells proliferated slowly and seemed to be quiescent. 

A similar population of VSEL-like stem cells was much more abundant in cell cultures of recurrent ovarian cancer ascites, where they represent 86% of all cells and were the predominant population of cells (Table 1 and Figure 2). In contrast to the other two types of cell cultures, these yellow, shining VSEL-like stem cells from ascites (Figure 2A–C) were highly proliferating and formed tumour-like structures (Figure 2D–F), which were comparable to those in hESC and normal ovary cell cultures (Figure 1C,H,I). They were attached to the dish bottom and proliferating, formed cell protrusions like “seeds” and lost the yellow shine (Figure 2G,I). In some places, we clearly observed that these VSEL-like stem cells spontaneously grew into spheroids, which were characteristic of cancer stem cells (Figure 2H,I), or larger oocyte-like cells resembling primitive oocytes with diameters of approximately 60 μm (Figure 2J–L).

**Figure 3:**
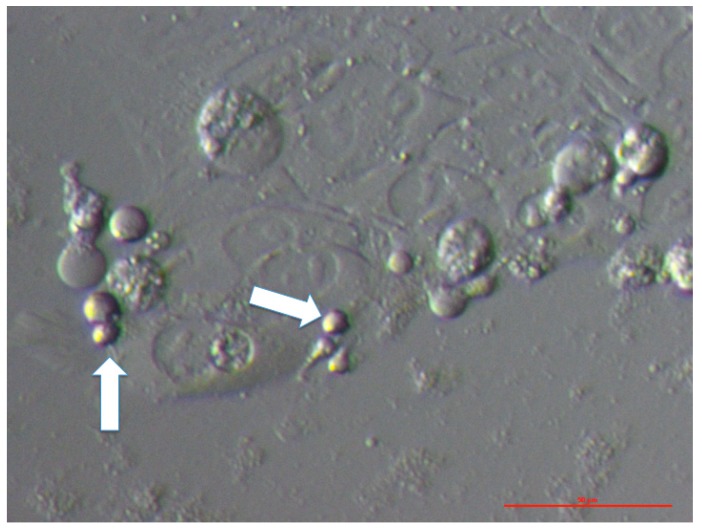
VSEL-like stem cells (arrows) with diameters of up to 5 μm among the cells of an embryo at the blastocyst stage that spontaneously separated after a biopsy for preimplantation genetic diagnosis in the in vitro fertilization programme. Embryonic cells began to attach to the dish bottom and spread. Red bar: 50 μm.

**Figure 4:**
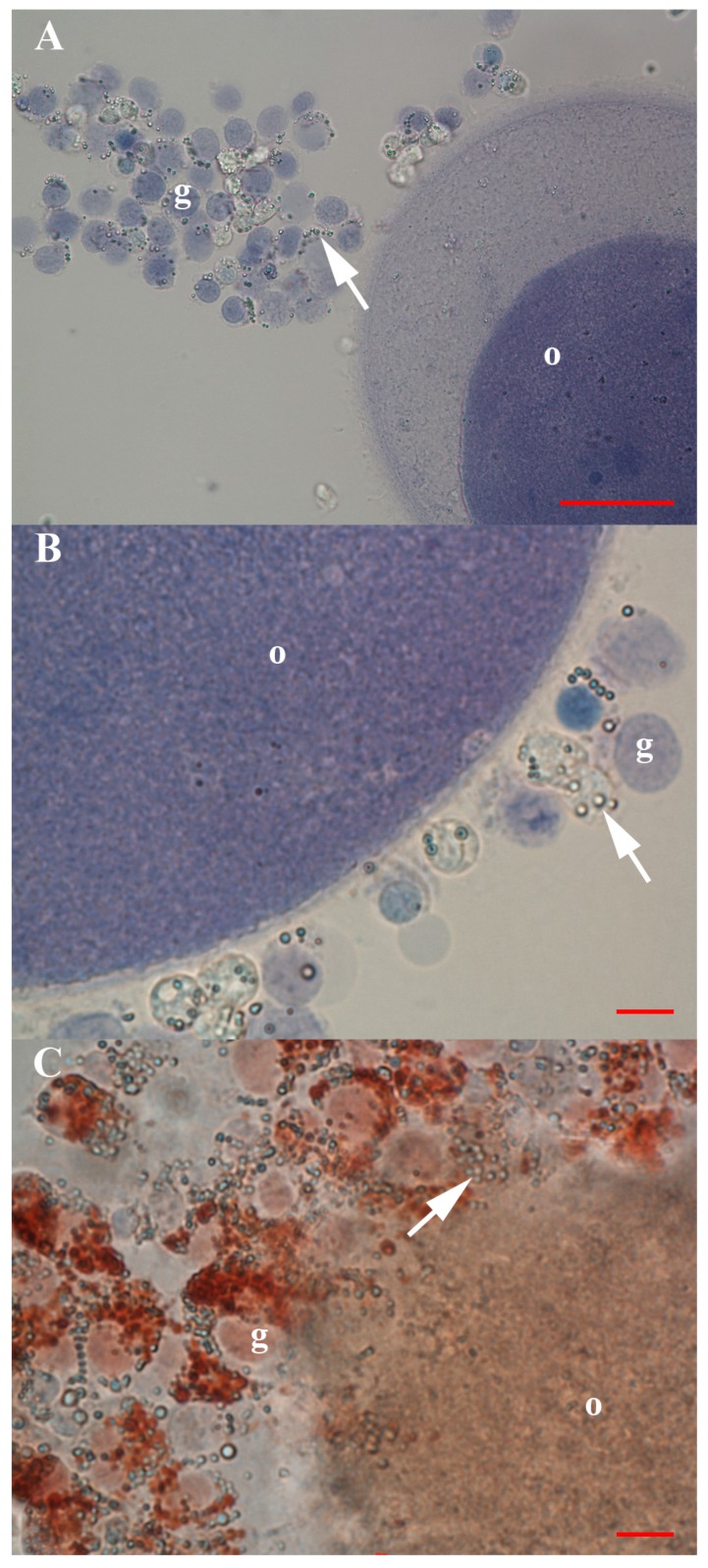
Oocytes that were isolated from the normal ovarian tissue prior to the cell culture and surrounded by follicular (granulosa) cells and a similar population of VSEL-like stem cells (arrows) with diameters of up to 5 μm among them. (**A**) Dead oocyte surrounded by an uneven zona pellucida and granulosa cells (live and dead) attached to it after Trypan Blue staining; among granulosa cells, there was a population of VSEL-like stem cells (arrow). (**B**) Dead oocyte surrounded by a very thin zona pellucida and granulosa cells (live and dead) attached to it after Trypan Blue staining; among granulosa cells, there was a population of VSEL-like stem cells (arrow). (**C**) Live oocyte surrounded by a mass of granulosa cells (live and dead) after Neutral Red staining; among granulosa cells, there was a population of VSEL-like stem cells (arrow). Legend: o—oocyte; g—granulosa cells; blue—dead cells after Trypan Blue staining; red—live cells after Neutral Red staining. Red Bar: 10 μm for B, C; 50 μm for A.

Small VSEL-like stem cells with diameters of up to 5 μm were present between the cells of an embryo at the blastocyst stage, which spontaneously separated after the biopsy for preimplantation genetic diagnosis in the in vitro fertilization programme (Figure 3). In addition, the oocytes that were isolated from the normal ovarian tissue prior to cell culture were surrounded by follicular (granulosa) cells, among which, a similar population of VSEL-like stem cells with a diameter of up to 5 μm was observed (Figure 4); these small cells were mostly alive (72%) after retrieval. 

In a parallel cell culture of human adult dermal fibroblasts, we did not observe any VSEL-like stem cells, development of oogonial- and oocyte-like cells or formation of spheroid-like structures (Supplemental Figure S1).

### 3.2. Positivity of VSEL-Like Stem Cells, Spheroids and Oocyte-Like Cells for the Germinal Lineage-Related Markers DDX4 and PRDM14

**Figure 5:**
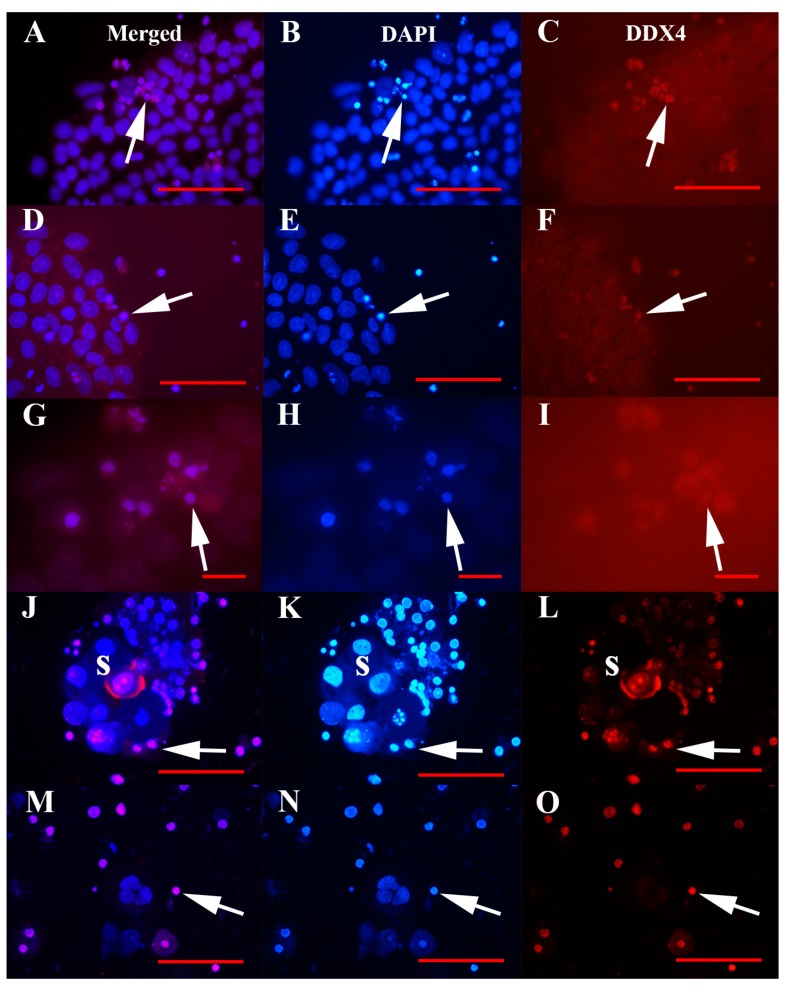
DDX4-positive VSEL-like stem cells with diameters of up to 5 μm in cell cultures, as revealed using immunofluorescence. (**A**–**I**) Human embryonic stem cells (hESCs), and (**J**–**O**) recurrent ovarian cancer ascites cell culture with spheroids. Fluorescence microscope. Legend: s—spheroid; red fluorescence—DDX4-positivity; blue fluorescence—genetic material after DAPI staining. Red bar: 10 μm for G–I; 50 μm for A–F, J–O.

VSEL-like stem cells with diameters of up to 5 μm were real cells and not cell debris, as shown in Figure 5. Their consistent nuclei filled the whole cell volumes and were strongly stained blue by DAPI. In all types of cell cultures, these VSEL-like stem cells stained red for the germinal lineage-related marker DDX4 (VASA), especially in hESCs (Figure 5A–I) and ascites (Figure 5J–O) cell cultures, as revealed using immunofluorescence. Among the VSEL-like stem cells with compact nuclei, there was a proportion of senescent cells with fragmented genetic material (Figure 5G,H). In hESC cultures, these VSEL-like stem cells were the only type of cells that were DDX4-positive, while the predominant cells that were spread on the culture dish bottom were negative. In ascites cell cultures, a proportion of cells in larger, well-developed spheroids expressed DDX4 protein; this protein was expressed both in the cell nuclei and cytoplasm (Figure 5J,L).

**Figure 6:**
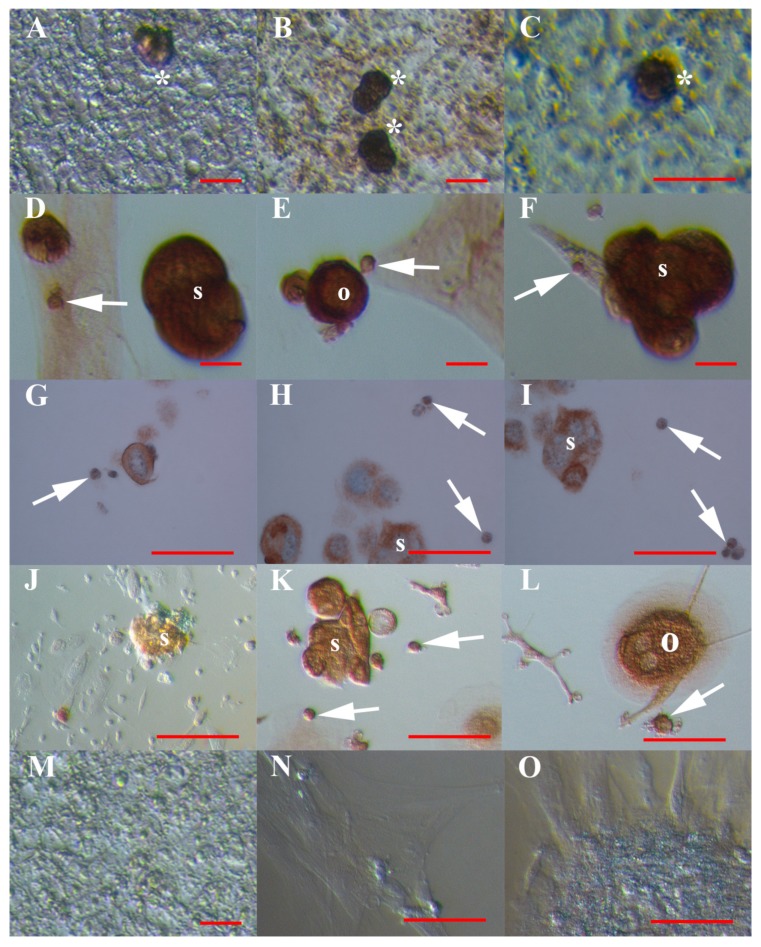
Positive (brown) staining of VSEL-like stem cells (arrows) with diameters of up to 5 μm, cell clusters (asterisks), spheroids (s) and oocyte-like cells (o) for germinal lineage markers using the DAB method of immunocytochemistry. (**A**–**C**) DDX4-positive cell clusters in hESC cell cultures; (**D**,**F**) DDX4-positive spheroids and VSEL-like stem cells and (**E**) DDX4-positive oocyte-like cells and VSEL-like stem cells in cultures of ascites cells from recurrent ovarian cancer; (**G**–**I**) DDX-4-positive larger cells in spheroids and VSEL-like stem cells in ascites of recurrent ovarian cancer; (**J**,**K**) PRDM14-positive spheroids and (**L**) PRDM-14-positive oocyte-like cell with a diameter of around 60 μm and surrounded by a structure resembling the zona pellucida of human oocytes, and VSEL-like stem cells; and (**M**–**O**) negative controls. Inverted microscope (Hoffman illumination). Red bar: 10 μm for A–**F**, M; 50 μm for G-I, K, L, N, O; 100 μm for J.

These observations were further confirmed by another method of immunocytochemistry, DAB, which showed strong DDX-4 positivity of the VSEL-like stem cells and clusters of these cells in hESC (Figure 6A–C) and ascites (Figure 6D–I) cell cultures. In ascites cell cultures, spheroids (Figure 6D,F) and oocyte-like cells (Figure 6E) strongly expressed DDX4 protein. Moreover, the VSEL-like stem cells, spheroids and oocyte-like cells in ascites cell cultures expressed another germinal lineage-related marker, PRDM14 (Figure 6J–L), which was expressed in their cytoplasm; in some oocyte-like cells, the zona pellucida-like structure was developed (Figure 6L). The human adult dermal fibroblasts were not positively stained for DDX4 and PRDM14 markers of germinal lineage.

The spheroids were also embedded in agarose gel, embedded in paraffin, and stained with haematoxylin-eosin (HE) staining and immunostaining for DDX-4 expression. These staining protocols confirmed the presence of two predominating types of cells in spheroids: larger cells and smaller, VSEL-like stem cells with diameters of up to 5 μm, which both expressed blue stained nuclei and were positively stained for the DDX4 protein (Figure 6G–I). In larger cells, only the cell cytoplasm was positively stained for the DDX4 marker of the germinal lineage.

### 3.3. Positivity of Small VSEL-Like Cells for CD133 and Coexpression with the Germinal Marker DDX4

**Figure 7:**
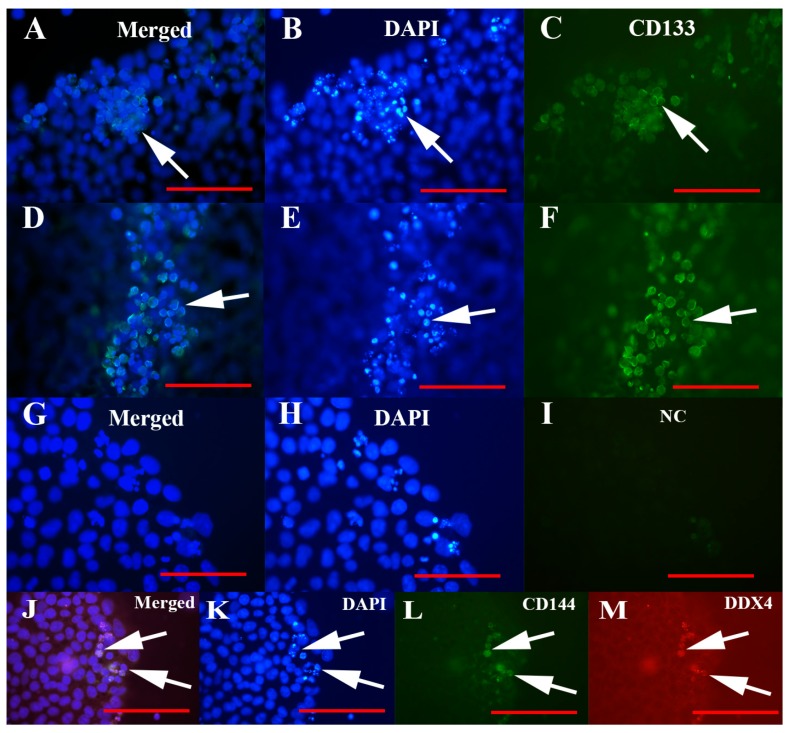
CD133 expression of VSEL-like stem cells in human embryonic stem cell (hESC) cultures and its coexpression with the DDX4 marker. (**A**–**F**) Expression of the CD133 marker, (**G**–**I**) negative control, and (**J**–**M**) coexpression of the markers CD133 and DDX4. Fluorescence microscope. Legend: green fluorescence—positivity for CD133; red fluorescence—positivity for DDX4; blue fluorescence—genetic material stained blue after DAPI staining. Red bar: 50 μm.

The population of VSEL-like stem cells in all types of cell cultures, including hESCs, expressed a stem cell-related marker, CD133, as revealed using immunocytochemistry (Figure 7). This marker was expressed at the cell surface of VSEL-like stem cells (Figure 7A,C,D,F).

Double immunostaining of cell cultures for expression of CD133 and DDX4 markers showed that the same population of VSEL-like stem cells, small cells with diameters of up to 5 μm, were positively stained for both markers (Figure 7J–M).

### 3.4. MACS Sorting of CD133+ Cells

**Figure 8:**
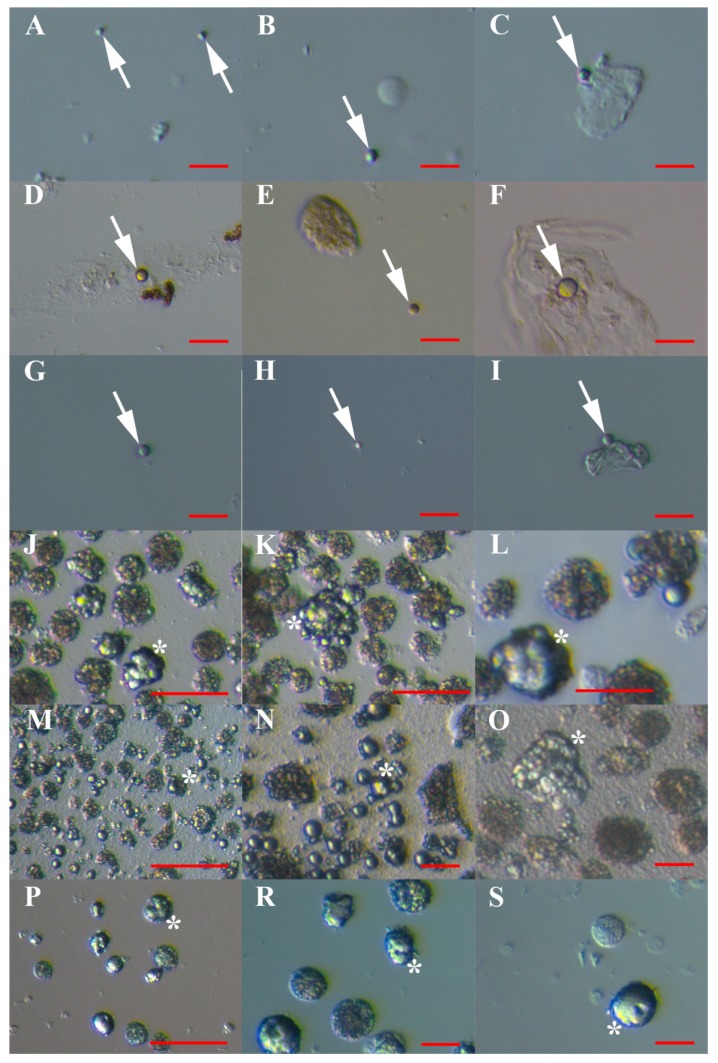
VSEL-like stem cells (arrows) with diameters of up to 5 μm and cell clusters (asterisk) after CD133-based magnetic-activated cell sorting (MACS). (**A**–**I**): Just after MACS sorting: human embryonic stem cells (hESCs) (A–C), normal ovary cells (D–F) and recurrent ovarian cancer ascites (G–I); (**J**–**S**) after artificial activation with valproic acid and FSH: human embryonic stem cells (hESCs) (J–L), normal ovary cells (M–O), and recurrent ovarian cancer ascites (P–S). Inverted microscope (Hoffman illumination). Red bar: 10 μm for A–I, L, N, O, R, S; 50 μm for J, K; 100 μm for M, P.

A similar population of VSEL-like stem cells with diameters of up to 5 μm predominated, and were sorted on the basis of expression of the stem cell-related marker CD133 from all types of cell cultures: hESCs (Figure 8A–C), normal ovary cells (Figure 8D–F), and ascites in recurrent ovarian cancer (Figure 8G–I). The VSEL-like stem cells represented more than 50% of the sorted cells in all cell cultures: 90.0% in hESCs, 56.4% in the ovary, and 64.3% in ascites. The proportion of VSEL-like stem cells was significantly higher in the CD133+ cell population, sorted from hESCs, than from ovarian (*p* < 0.01) and ascites cell cultures (*p* < 0.01), as revealed by the chi-square test. They appeared as individual cells (Figure 8A,B,D,E,G,H) or were attached to other types of cells (Figure 8C,F,I). These cells seemed to be quiescent and did not proliferate, except those from ascites cultures, which proliferated and spontaneously formed spheroids or grew into larger, oocyte-like cells after sorting. In the CD133− cell fraction (negative selection), almost no VSEL-like stem cells were observed, with the exception of rare cells (<1%) that were attached to other types of cells. 

### 3.5. Artificial Expansion of VSEL-Like Stem Cells after CD133-based MACS Sorting Using Valproic Acid and FSH

After MACS sorting, the VSEL-like stem cells started to intensely proliferate from two to three months after exposure to valproic acid and FSH, while this was not observed in a control cell culture without the addition of valproic acid and FSH. In all types of cell cultures—hESCs (Figure 8J–L), normal ovary cells (Figure 8M–O), and ascites cells (Figure 8P–S)—the sorted VSEL-like stem cells started to intensely proliferate and formed larger cell clusters resembling tumour-like structures. 

A proportion (up to 15%) of VSEL-like stem cells grew to larger, oogonial-like cells with diameters of approximately 10 μm. The cell cultures were maintained for up to 8 months, and in all cell cultures, the population of activated VSEL-like stem cells was proliferating, while the rest of the cells were degenerating during this time, including hESCs. After 8 months of culture, 41% of cells were still alive. 

### 3.6. Differentiation of Sorted and Artificially Activated CD133+ VSEL-Like Stem Cells into Adipogenic, Osteogenic and Neural Lineages

**Figure 9:**
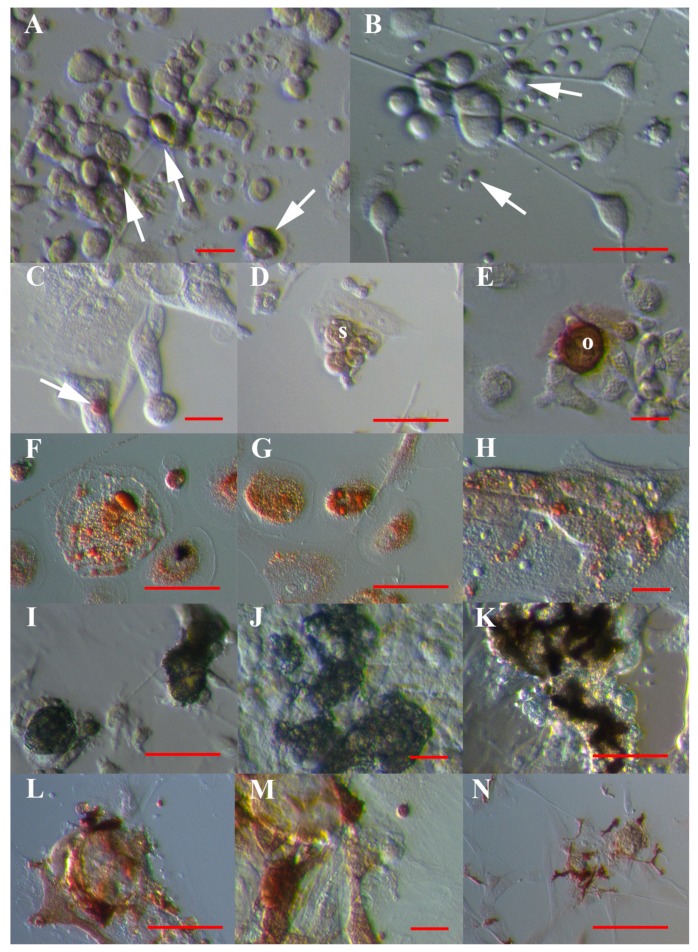
In vitro differentiation of VSEL-like stem cells (arrows) with diameters of up to 5 μm sorted using MACS according to established protocols. (**A**,**B**) VSEL-like stem cells from recurrent ovarian cancer ascites (arrows) attached to the dish bottom, losing the yellow “shining” and spontaneously developing different phenotypes with protrusions. (**C**) VSEL-like stem cells in a hESC cell culture expressing the pluripotency-related marker SSEA-4 (brown), as revealed by the DAB procedure. (**D**) SSEA-4-positive (brown) spheroid and (**E**) SSEA-4-positive (brown) oocyte-like cells developed from small stem cells in ascites cell culture. (**F**–**H**) Differentiation into adipogenic lineage; lipid droplets were stained red after Oil Red O staining: normal ovary (F), ascites (G) and hESCs (H). (**I**–**K**) Dedifferentiation into osteogenic lineage with calcium deposits, which were stained black after von Kossa staining: normal ovary (I), ascites (J) and hESCs (K). (**L**–**N**) Differentiation into a neural lineage after S100 staining (brown): normal ovary (L), ascites (M) and hESCs (N). Inverted microscope (Hoffman illumination). Legend: s—spheroid; o—oocyte-like cell. Red bar: 10 μm for A–C, E, H, J, M; 50 μm for D, F, G, I, K, L; 100 μm for N.

After MACS sorting, VSEL-like stem cells with diameters of up to 5 μm attached to the dish bottom, lost their yellow shine and began to spontaneously grow into larger cells, resulting in spheroids (ascites cultures) or cells with different phenotypes (Figure 9A,B). After sorting, a proportion of VSEL-like stem cells (Figure 9C), spheroids (Figure 9D) or growing cells (Figure 9E) expressed the stem cell marker SSEA-4, as revealed by immunocytochemistry (DAB procedure).

When exposed to established differentiation media, the sorted cells/cell cultures of all types—hESCs, normal ovary cells, and ascites—developed into adipogenic (Figure 9F–H), osteogenic (Figure 9I–K) and neural lineages (Figure 9L–N), which were confirmed using Oil Red O and von Kossa staining and immunocytochemistry for S100 marker expression. Taking into account all cell lines, 49.1% of activated cells differentiated in vitro into the neural lineage, 36.1% of the cells into the adipogenic lineage and 21.4% into the osteogenic lineage. A significantly higher proportion of cells differentiated into the neural lineage than in the adipogenic (*p* < 0.01) and osteogenic lineages (*p* < 0.01), as revealed by the chi-square test. On the other hand, the cell culture of human adult dermal fibroblasts did not differentiate in vitro into either of these cell lines. Similarly, the population of CD133− cells and non-activated CD133+ cells did not differentiate into the neural, adipogenic or osteogenic lineages. 

### 3.7. qPCR Analysis of the Expression of the CD133 and DDX4 Genes in Cell Cultures

Preliminary genetic analyses confirmed the expression of *CD133* and *DDX4* in all types of cell cultures: hESCs, normal ovary and ascites after MACS-sorting (Table 2). Because of the small number of samples, a statistical analysis was not performed. A more than two-fold change in gene expression compared to the normal ovary sample was considered up- or down-regulation of a specific gene. In ascites and hESC cultures, the expression of the *CD133* gene was up-regulated compared to cell cultures from normal ovaries; the highest expression of the *CD133* gene was found in hESCs (Table 2). This gene was also expressed in positive control, colon cancer HT-29 cells at approximately the same level as in ascites cells, while it was not expressed in a negative control, adult human fibroblasts. Similarly, *DDX4* expression was up-regulated in hESCs and ascites cells compared to cell cultures from normal ovaries, while it was not expressed in HT-29 cells and fibroblasts (Table 2). In ovarian samples, normal ovary and ascites, the expression of *CD133* and *DDX4* genes was approximately the same, while in hESCs, the expression of *DDX4* was lower than the expression of *CD133* (Table 2).

## 4. Discussion

The results of this research showed, for the first time, the presence of a similar population of stem cells with diameters of up to 5 μm resembling very small embryonic-like stem cells (VSELs) and expressing stem cell and germinal lineage-related markers in cell cultures of hESCs, normal ovary cells and ascites in recurrent ovarian cancer. These cells were successfully sorted from all cell cultures based on the expression of the stem cell-related marker CD133. Although naturally quiescent after sorting, they highly proliferated and formed cell clusters resembling tumour-like structures after exposure to valproic acid (VPA) and FSH, and were successfully differentiated in vitro into adipogenic, osteogenic and neural lineages after sorting.

Our data confirmed the stemness of VSEL-like stem cells sorted from hESCs, normal ovary and recurrent ovarian cancer in addition to different aspects. These cells expressed consistent nuclei that filled almost the entire cell volume, expressed the stem cell-related markers CD133 and SSEA4, were highly proliferating after artificial activation with valproic acid and FSH, and after sorting, differentiated into other types of cells of adipogenic, osteogenic and neural lineages using established differentiation protocols. In this study, valproic acid was used for the first time to activate VSEL-like stem cells in vitro from ovaries that are usually quiescent after sorting and are highly efficient. Some other studies showed that valproic acid, a fatty acid derivative, was efficient in ex vivo expansion of other types of stem cells, such as haematopoietic stem cells from umbilical cord blood [54] and placenta-derived mesenchymal/stromal stem cells [55]. Otherwise, it is an important FDA-approved drug component with several beneficial effects and of great interest for the treatment of different types of cancers [56]. As a medication, it is mostly used to treat seizure disorders, mental/mood conditions, such as manic phase of bipolar disorder [57], and to prevent migraine headaches [58].

Furthermore, we found that VSEL-like stem cells from the ovaries may exhibit a FSH-R3 receptor and that FSH may act on VSELs directly to modulate the ovarian stem cells and augment the oogenesis/folliculogenesis and primordial follicle assembly [59,60,61], which may explain the potential role of FSH in additionally enhancing the in vitro proliferation of VSEL-like stem cells after MACS sorting in our study. The expansion of small VSEL-like cells in vitro after their sorting will help further analyses of these stem cells and develop new cell therapies in the future.

VSEL-like stem cells with diameters of up to 5 μm expressed the germinal lineage markers DDX4 and PRDM14, and the marker DDX4 was coexpressed with the marker CD133. Therefore, these cells might be very small embryonic-like stem cells, which are known to strongly express the stem cell-related marker CD133 [35,36] and are proposed to persist from the embryonic period of life [19,20]. In our study, these VSEL-like stem cells were also sorted from hESC cell cultures, which may confirm their presence in the human preimplantation embryos and further confirmed that they were very small embryonic like-stem cells. These cells were also sorted from cell cultures from normal ovaries at lower numbers but were the predominating, highly proliferating cells in ascites of recurrent ovarian cancer. In cell cultures from recurrent ovarian cancer ascites, these VSEL-like stem cells were highly proliferating in vitro without artificial activation, thus indicating that in cancers, these cells are highly active, while in a normal ovary, they are not. This finding means that the cancer somehow activates these VSEL-like stem cells, which are otherwise quiescent. In ascites cell cultures, the proportion of VSEL-like cells was significantly greater than in cell cultures of normal (non-malignant) ovary cells. The VSEL-like stem cells formed spheroids and tumor-like structures in vitro and expressed the gene *CD133* at a higher level than VSEL-like stem cells from normal ovary cells.

They may be involved in the manifestation of ovarian cancer, as previously confirmed in borderline ovarian cancer [51], and this may explain the high expression of the marker CD133 in different cancers, including the ovarian tumours found by other studies [37,38,39,40]. In a recent study, Clarkson et al. sorted a similar population of small DDX4/ALDH1-positive cells from ovarian cell cultures using fluorescence-activated cell sorting (FACS) using the combined activity of these two markers; they found three different populations of positive cells, two of which were small, ranging from 3 to 10 μm, and developed into primitive follicle-like structures, including oocyte-like cells [62]. Interestingly, they found coexpression of the germinal lineage-related marker DDX4 with an important stem/progenitor cell marker (ALDH1) in these cells. ALDH1 is also expressed in ovarian cancer stem cells [63] and is associated with poor prognosis in ovarian cancer [64,65], which indicates a possible connection of small stem cells with ovarian cancer.

We suggest that small stem cells, namely VSEL-like stem cells, as the original embryonic stem cells present in human preimplantation embryos and embryonic stem cell cultures (hESCs), persist in adult ovaries at the pre-oogonial stage and are involved in the manifestation of ovarian cancer, including recurrent ovarian cancer with ascites.

## Figures and Tables

**Table 1:** Proportion of VSEL-like stem cells in different cell cultures prior to MACS-sorting.

	Types of Cell Cultures				Statistical Significance
	hESCs	Normal Ovaries	Ascites	Fibroblasts (Negative Control)	
**Mean proportion of VSEL-like stem cells/field**	35% ^ῶ^ (min. 10%–max. 50%)	17% * (min. 0%–max. 67%)	75% *^ῶ^ (min. 33% –max. 75%)	0%	*^ῶ^ *p* < 0.0001
**Proportion of VSEL-like stem cells/counted cells**	32% ^⌘^	10% ^υ^	86% ^υ^^⌘^	0%	^υ^^⌘^*p* < 0.01

*^ῶ^ t-test with statistical significance set at *p* < 0.05. ^υ^**^⌘^** Chi-square test with statistical significance set at *p* < 0.05.

**Table 2:** Preliminary qPCR analysis of the expression of the *CD133* and *DDX4* genes in cell cultures from normal ovaries, ascites of recurrent ovarian cancer and human embryonic stem cells (hESCs) after sorting, and the colon cancer cell line HT-29 (positive control) and human adult dermal fibroblasts (negative control). Gene expression was normalised relative to a normal ovary. Because of the small number of samples, the statistical analysis was not performed. A more than two-fold change in gene expression compared to the normal ovary sample was considered up- or down-regulation of a specific gene. *Legend*: ND-not detected; gene was not expressed.

Cell Cultures	Gene Expression Level (± Standard Deviation)	
	*CD133*	*DDX4*
Normal ovary	1.00 ± 0.10	1.00 ± 0.16
Ovarian cancer ascites	8.58 ± 0.80	9.55 ± 2.15
hESCs	12.00 ± 1.21	3.61 ± 0.67
HT-19	8.20 ± 0.74	ND
Fibroblasts	ND	ND

## Supplementary Materials

The following are available online at https://www.mdpi.com/2073-4409/8/7/706/s1, Figure S1: Cell culture of human adult dermal fibroblasts (negative control); Figure S2: Labeling check of CD133+ cell population after MACS sorting.
