# Supplementary material for: Similar Population of CD133+ and DDX4+ VSEL-Like Stem Cells Sorted from Human Embryonic Stem Cell, Ovarian, and Ovarian Cancer Ascites Cell Cultures: The Real Embryonic Stem Cells?
Source: Cells. 2019 Jul 11;8(7):706. doi: 10.3390/cells8070706 (PMC6678667; doi:10.3390/cells8070706)
Supplement: Supplementary file 1 [file cells-08-00706-s001.pdf]

Supplemental Material

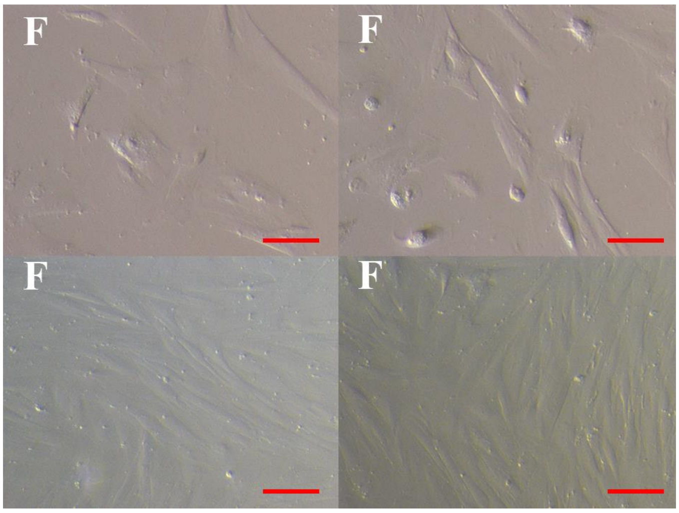

Supplemental Figure S1: Cell culture of human adult dermal fibroblasts (negative control). *Legend*: F-fibroblasts. Red bar: 50 µm.

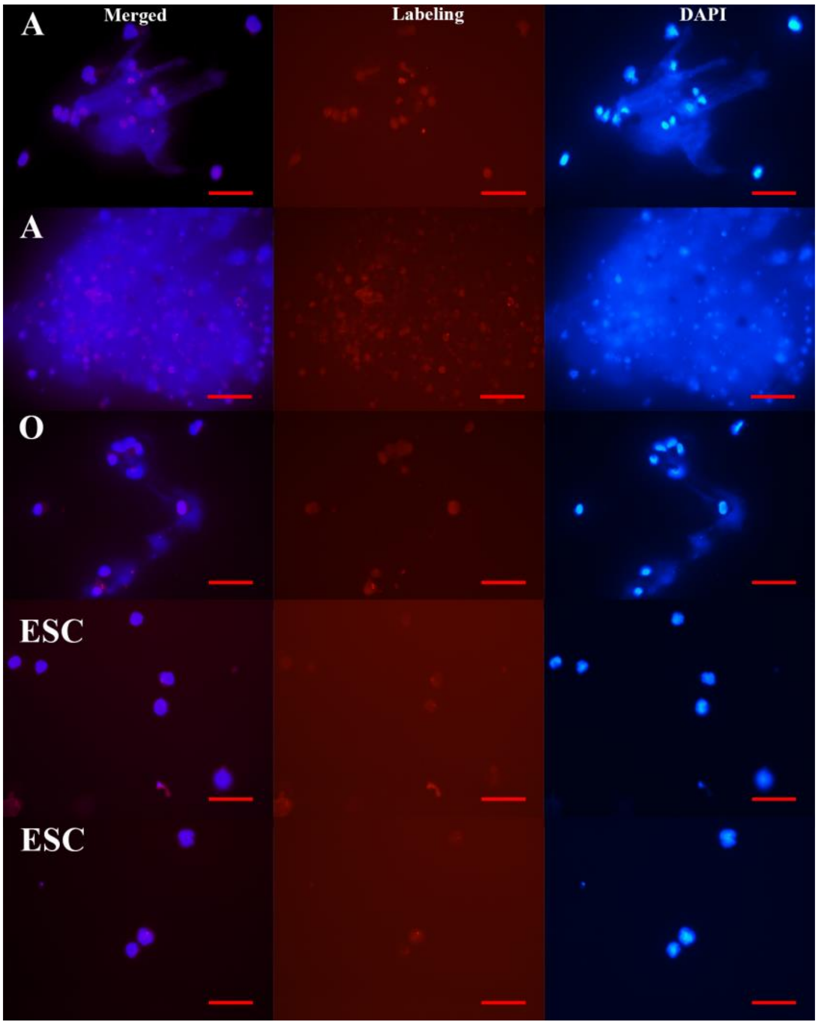

Supplemental Figure S2: Labeling check of CD133+ cell population after MACS sorting. Legend: A-ascites; O-ovary; ESC-embryonic stem cells. Red bar: 10 µm.
